# Single-trial classification of awareness state during anesthesia by measuring critical dynamics of global brain activity

**DOI:** 10.1038/s41598-019-41345-4

**Published:** 2019-03-20

**Authors:** Leandro M. Alonso, Guillermo Solovey, Toru Yanagawa, Alex Proekt, Guillermo A. Cecchi, Marcelo O. Magnasco

**Affiliations:** 1 Laboratory of integrative neuroscience, The Rockefeller University, New York, NY 10065 USA; 2 Present Address: Volen Center for Complex Systems, Department of Biology, Brandeis University, Waltham, MA 02454 USA; 3 Instituto del Cálculo, FCEyN, Universidad de Buenos Aires, (C1428EGA), Buenos Aires, Argentina; 4 Laboratory for Adaptive Intelligence, Brain Science Institute, RIKEN, Saitama, 351-0198 Japan; 5 Anesthesiology and Critical Care, University of Pennsylvania, Philadelphia, PA 19104 USA; 6 IBM, Thomas J. Watson Research Center, Yorktown Heights, NY USA

**Keywords:** Dynamical systems, Network models

## Abstract

In daily life, in the operating room and in the laboratory, the operational way to assess wakefulness and consciousness is through responsiveness. A number of studies suggest that the awake, conscious state is not the default behavior of an assembly of neurons, but rather a very special state of activity that has to be actively maintained and curated to support its functional properties. Thus responsiveness is a feature that requires active maintenance, such as a homeostatic mechanism to balance excitation and inhibition. In this work we developed a method for monitoring such maintenance processes, focusing on a specific signature of their behavior derived from the theory of dynamical systems: stability analysis of dynamical modes. When such mechanisms are at work, their modes of activity are at marginal stability, neither damped (stable) nor exponentially growing (unstable) but rather hovering in between. We have previously shown that, conversely, under induction of anesthesia those modes become more stable and thus less responsive, then reversed upon emergence to wakefulness. We take advantage of this effect to build a single-trial classifier which detects whether a subject is awake or unconscious achieving high performance. We show that our approach can be developed into a means for intra-operative monitoring of the depth of anesthesia, an application of fundamental importance to modern clinical practice.

## Introduction

Over the last century the invention of general anesthesia transformed modern medicine by enabling highly invasive surgeries and diagnostic procedures to be performed while the patient is rendered unconscious. Since the 1930’s it has been proposed that the anesthetized state can be monitored using electroencephalography (EEG)^1^. Exactly how the EEG ought to be monitored and interpreted to assure that, on the one hand, the patient is not awake during surgery and, on the other hand, not overdosed on anesthetics is still not clear.

Many different measures have been applied to quantify the effects of anesthetics on brain activity. Most commonly deployed measures include the spectral characteristics of the EEG^2^. Indeed, many anesthetics such as propofol elicit the canonical slow waves, also associated with slow wave sleep^1^ and anteriorization of alpha oscillations^2–4^. Yet, other anesthetics, most notably ketamine, are not reliably associated with slowing of the EEG^5^ or the anterior shift in alpha oscillations^6^. Furthermore, even at a fixed anesthetic concentration, spectral characteristics of local field potentials recorded from the thalamus and cortex fluctuate stochastically among different discrete states^7^. Other EEG-based measures of anesthetic depth include the bispectral index^8^. Yet, bispectral index is also not reliably altered by ketamine^9^ and other anesthetics^8^. More recent attempts at quantifying the effects of anesthetics on brain activity focused on functional connectivity between different brain areas. This promising approach identified that frontoparietal connectivity is suppressed by mechanistically distinct anesthetics that include ketamine, propofol and sevoflurane^6,10^. Indeed, as consciousness is thought to be an emergent phenomenon arising out of the interactions between different brain areas, it seems likely that a robust and theoretically sound measure of anesthetic depth should take into account the interactions between signals emitted by different parts of the cortex. Consistent with this line of reasoning Massimini and colleagues demonstrated that loss of consciousness associated with sleep^11,12^, anesthesia^13^, and brain injury^14^ result in decrease in the complexity of responses elicited by transcranial magnetic stimulation. Yet, it is unclear what dynamical features of brain activity result in the disruption of functional connectivity and loss of complexity of evoked responses.

One possible explanation for loss of connectivity and complexity of responses observed in the unconscious state is that in order to exhibit consciousness the brain must operate in a critical regime similar to phase transitions in physics, given several computational desirable features of such states represented by the statistics of the thermodynamic variables^15^. Evidence for *statistical* criticality is based on the observation that various aspects of neuronal activity such as avalanches observed in local field potentials and action potentials in tissue preparations and in animal models^16,17^, as well as magneto-encephalography (MEG) and electro-corticography (ECoG) in human subjects^18,19^, exhibit long tailed-distributions well approximated by power laws. More recently, the *dynamical* aspect of criticality has been brought into focus, as a similarly desirable feature not fully captured by steady-state statistics such as avalanche size distributions^20–22^; a perturbation in an extended dynamical system that is close to a critical point will neither decay nor explode, thus allowing for long range communication across the entire system. This will manifest as increase in functional connectivity and the complexity of responses. In contrast, if the system is far from criticality (therefore stable), perturbations damp out and no information integration takes place beyond the characteristic time scale which characterize the damping. This will result in the apparent loss of functional connectivity and loss of complexity of responses.

The *dynamically* critical regime provides important functional benefits; quantities such as dynamic range and information transmission are optimized near criticality^23^. If indeed dynamical criticality is a useful feature of brain activity, stability of neuronal dynamics ought to be modulated by the behavioral state of the subject. When the brain is awake and displaying complex statistical behavior its dynamical state ought to be close to a bifurcation point; marginally stable modes contribute to long range interactions across the system. Conversely when higher-order functions associated with wakefulness have been diminished and eventually completely shut down by anesthesia, brain dynamics should exhibit more stability. In other words, anesthesia induction should lead to stabilization of brain dynamics.

To test these hypotheses, we fitted vector autoregressive (VAR) models to electrocorticography (ECoG) recordings obtained by placing an electrode grid covering much of the cerebral hemisphere in non-human primates^24^ and in human subjects undergoing epilepsy surgery^25^. These studies confirmed that loss of consciousness was associated with the stabilization of brain dynamics. In the awake state, the dynamical global modes of brain activity inferred using the VAR models hover in the vicinity of the critical point between stability and instability. When consciousness is lost, global modes become more stable^24,25^. Remarkably, we observed stabilization with conventional anesthetics such as propofol and those that elude the traditional analysis methods such as ketamine^24,25^. Thus, loss of *dynamical criticality* may offer a unified view which explain both loss of functional connectivity and complexity of the evoked responses in the unconscious brain.

Here, we build upon our previous observations to determine the reliability of stabilization of brain dynamics as a means of distinguishing activity emitted by the brain in the conscious and the unconscious states. Our previous studies have shown statistically significant differences in stability for each individual by aggregating a fair amount of data. However this does not permit looking at a few seconds of activity from one patient and state whether the patient is awake or unconscious, because the baseline for each individual is unknown. In this work we develop a single-trial classifier which aims to determine the state of the subjects using short temporal snippets of the ECoG recordings. We build vectors based on the dynamical stability (DS) of the ECoG signals and train support vector machines (SVM) to classify the subjects’ states. Our results suggest that one may be able to build a baseline-free classifier using measures based on dynamical stability.

## Methods

### Use of experimental animals

All surgical procedures and experiments were performed in accordance with the experimental protocols (No. H24-2-203(4)) approved by the RIKEN ethics committee and the recommendations of the Weatherall report, *The use of non-human primates in research*. Implantation surgery was performed under sodium pentobarbital anesthesia, and all efforts were made to minimize suffering. No animal was sacrificed in this study. Overall care was managed by the Division of Research Resource Center at RIKEN Brain Science Institute. The animals were housed in a large individual enclosure with other animals visible in the room, and maintained on a 12:12-h light:dark cycle. The animal was given food (PS-A; Oriental Yeast Co., Ltd., Tokyo, Japan) and water ad libitum, and also daily fruit/dry treats as a means of enrichment and novelty. The animal was occasionally provided toys in the cage. The in-house veterinary doctor checked the animal and updated daily feedings in order to maintain weight.

### Subjects and data acquisition

Data from four male monkeys were collected at the Laboratory for Adaptive Intelligence, Brain Science Institute, RIKEN. Electrocorticographic (ECoG) recordings were sampled at 1 kHz from an array consisting of *N* = 128 electrodes covering both full hemispheres. In this study we analyze a total of 16 experiments each consisting of reversible induction of anesthesia starting from the awake state. A more detailed description of the experiments can be found in [Nagasaka *et al*., 2011; Yanagawa *et al*., 2013]. The dataset consists also of video recording which allow the visualization of the behavioral assessments of the level of consciousness. These video recordings were used to precisely associate the state of consciousness of the animal and the contemporaneously recorded neuronal activity.

### Description of the dataset

A total of 12 anesthetic inductions were performed using ketamine medetomidine (KM) doses. Four anesthetic inductions were performed with propofol (P). Ketamine medetomidine inductions were performed by injecting the drugs intramuscularly, whereas propofol was administered intravenously. Each monkey received more than one anesthetic induction that were separated by at least 1 day. We labeled our subjects as (M1, M2, M3, M4). Our dataset consists of 4 sessions of KM for M1, 2 sessions of P for M1, 3 sessions of KM for M2, 3 sessions of KM for M3, 2 sessions of KM for M4 and 2 sessions of P for M4.

### Data processing

All channels were notch filtered to eliminate electrical line noise at 50 *Hz*, 100 *Hz* and 150 *Hz*. Then we applied a bandpass filter between 5 *Hz* and 500 *Hz*. Both notch and bandpass filters were implemented using the idealfilter function in MATLAB (MathWorks) to avoid phase shifts. These procedures were also performed using the python scipy function filtfilt yielding identical results.

### Stability analysis

The notion that the brain might be operating in a critical regime has been explored by many authors. Dynamical systems theory indicates that systems which are capable of performing computations should have a large number of modes with marginal stability. In such a scenario an arbitrary perturbation wont decay nor explode, thus allowing for information integration across the entire system. Therefore, it has been suggested that the brain might operate in a dynamically critical regime. A simple model exhibiting complex spatio-temporal dynamics was recently proposed by Magnasco *et al*. in which statistically critical behavior emerges due to dynamical instabilities^20^. Overall, theoretical considerations suggest that when the brain is awake its dynamical state is close to dynamical criticality, a state in which many dynamical degrees of freedom are neither unstable (they do not explode) nor stable (they do not decay) but straddling the interface between stability and instability. These marginally stable modes contribute to long range interactions across the brain. This leads us to associate wakefulness to dynamical marginality; conversely, the anesthetized brain should exhibit more damping in those modes that are associated to conscious function or cognition. If this view is correct, a measure of the dynamical stability of the system could then be used as a marker for depth of anesthesia^24,25^.

ECoG is a multivariate time series whose dynamical properties can be inferred using autoregressive models fitted independently to short time segments as described previously^26^. In order to test the notion that consciousness can be associated to a dynamical homeostatic mechanism, we assume locally linear dynamics in short temporal snipets of the recordings and fit vector autoregressive (VAR) models. This allows us to address changes in the stability properties of the fitted linear approximation as the concentration of anesthetics is increased. For a given temporal interval of duration *δ* we fit an order 1 VAR model. This is the simplest linear dynamical system that can be fit to a multivariate time series.

1 $${y}_{n+1}=A{y}_{n}+{u}_{n}$$

Here, ${y}_{n}\in {{\mathbb{R}}}^{N}$ is a multivariate time series that corresponds to the recorded activity in all *N* = 128 channels at time *t*_*n*_, $A\in {{\mathbb{R}}}^{N\times N}$ is the matrix to be estimated and *u*_*n*_ is assumed to be white noise. A comprehensive treatment of this model and its estimation can be found in Lütkephol^27^.

In this work we used a statistical modeling module for python called Statsmodels^28^. The idea behind the estimation procedure is that for a temporal segment containing *n* datapoints, one ends up with a system of *n* linear equations of the form 1. Then finding *A* under the assumption of uncorrelated noise can be cast as estimating the pseudoinverse of the matrix containing the *y*_*n*_ values. This is in turn performed by the standard python library numpy lstsq module^29^. Finally, this module is a python interface for the popular package LAPACK which contains several algorithms for performing singular value decompositions of large matrices^30^. The performance of our procedure relies ultimately on this standard and heavily tested library.

For each time window we obtain a matrix *A* using the algorithm described above. *A* governs the stability properties of the VAR model. In order to address changes in the dynamical stability of the fitted models we consider the distribution of eigenvalues of *A*. Since our underlying hypothesis corresponds to a continuum model we performed a transformation in order to obtain a correspondence between the eigenvalues of *A* and the timescales of the dynamics. Let ${\lambda }_{j}={\rho }_{j}{e}^{i\varphi }$ be the eigenvalue corresponding to the *j*-th mode, the frequency of the mode is given by ${f}_{j}=\frac{{\varphi }_{j}}{2\pi dt}$ while the growth rate (timescale) of the mode is given by ${\tau }_{j}=\frac{log({\rho }_{j})}{dt}$. Here $dt=\frac{1}{{S}_{f}}=0.001s$, where ${S}_{f}=1000\,Hz$ is the sampling frequency of the recordings.

Our previous results suggest that as the subjects become anesthetized the linear stability of the ECoG recordings exhibits significant stabilization which is efficiently quantified by non parametric statistical methods. Such stabilization effect was first reported in recordings performed in human patients with temporal lobe epilepsy while being anesthetized with propofol^25^. This finding was further supported by performing this analysis on the current dataset in monkeys^24^. This led us to associate loss of consciousness with stabilization of cortical activity. In this work we explore the possibility that such stabilization effect can be exploited to predict conscious activity.

### DS vectors

Dynamical stability (DS) is determined by the fitted distributions of eigenvalues. For a given temporal segment of duration Δ ≥ 500 *ms*, we build (DS) vectors based on this measure by coarse graining this distributions. We sample the distributions by fitting 10 equally spaced VAR(1) models using *δ* = 500 *ms* windows independently. Note that for choices of Δ ≤ 5000 *ms* the models fit overlapping data. Vectors are obtained by binning the distributions of eigenvalues in a 20 × 20 grid in the range $\tau =-250\frac{1}{s}$ to $\tau =25\frac{1}{s}$ and *f* = 4 *Hz* to *f* = 256 *Hz*. The frequency axis is in logarithmic scale base 2. The range and scales were chosen to highlight the differences between the awake distributions versus the anesthetized ones. About half of the total eigenvalues fall within this range. The vectors are obtained by flattening the distributions. This corresponds to horizontally stacking the row values of the grids yielding a vector with dimension 400.

### FFT vectors

In order to compare (DS) performance against a spectral method we build FFT vectors based only in spectral features of the data. For each channel, we compute the fast fourier transform of the data contained in an interval of duration Δ and keep the logarithm of the power *p* for frequencies smaller than 100 *Hz*; $v=log({p}_{f < 100Hz})$. A vector is defined as the average of *p* across channels. The dimension of the resulting vector then depends on the duration of the interval Δ. Performance is largely independent of this dimension.

### Data labeling

Each vector was labeled as awake or anesthetized by careful assessment of the experiment videos. For each experiment two temporal intervals were determined. The awake interval corresponds to resting with eyes closed condition, prior to any drug injection. The anesthetized intervals occur always after drug injection and were determined as the interval in between two behavioral assessments performed by the experimenters in which the subjects did not respond. Responsiveness assessments were in most cases tactile and in some cases the subjects were also perturbed with noise. Both intervals were further shortened by removing the first and last 30 seconds to decrease the chance of mislabeling. The interval durations are in general different across experiments ranging from 350 *s* to 1500 *s* for Ketamine Medetomidine. The anesthetized intervals are much shorter in the case of propofol ranging 130 *s* to 450 *s*. In all experiments the awake and anesthetized intervals are separated by at least 400 *s*.

### Surrogate tests

We applied three surrogate procedures to the ECoG recordings. Phase surrogation is a standard procedure which consists of Fourier transforming the data, randomizing the phases and transforming back. We applied this surrogation to each channel and used the surrogated data to build vectors. Staggering surrogation corresponds to desynchronizing the data locally. Each channel is shifted forward in time by a random value taken from a flat distribution of width $100\,\times \,\sqrt{12}\,ms$. This is performed every time an interval is selected for building a vector. Global staggering surrogation corresponds to the same procedure except that the shifts are determined only once and the same shifts are applied to all vectors of the same experiment. Global staggering surrogation was performed by taking the lags from a flat distribution of width 500 *ms*. All surrogate tests aim to disrupt global information across recording sites.

### Classification method

For each experiment we built a dataset by taking 500 equally spaced vectors on the awake interval and 500 equally spaced vectors in the anesthetized interval. For each dataset we also generated a surrogate dataset consisting of vectors built with surrogated data. The same procedure was also applied to obtain datasets for varying amounts of data Δ in order to test for performance as this parameter is varied. Depending on the choice of this parameter and the duration of the awake and anesthesia intervals of each experiment, adjacent vectors within an interval may be constructed with partially overlapping data.

We used support vector machines (SVM) for classification. SVMs in their simplest form are linear classifiers which are particularly efficient in high dimensional spaces and for which there are efficient training methods. In this work we implemented the machine learning python library scikit-learn^31^ to train SVMs with linear kernels for classifying whether a subject is awake or unconscious.

### Training and testing protocol

We test our classifiers on unseen data. Given a train dataset (T) and test dataset (P) to assess performance we proceed as follows. We take half the vectors on (T) *at random* to train the SVM and then ask the classifier to predict the full (P) dataset of a different subject. We then define the error as the number of wrong classifications in percent. Thus, one source of variability in the errors when this protocol is repeated comes from different choices for the training vectors.

## Results

**Figure 1:**
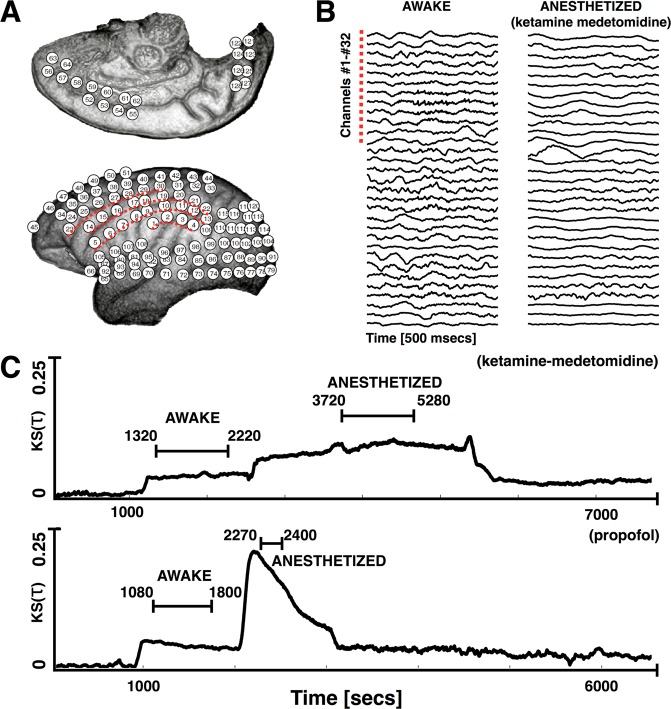
Changes in dynamical stability as subjects are induced into anesthesia. We computed the dynamical stability of VAR(1) models fitted to the ECoG recordings as the subjects underwent general anesthesia. The intervals labeled as *awake* and *anesthetized* were determined from responsiveness assesments performed during the experiments. (**A**) Electrode array used to fit VAR(1) models. (**A**) Data recorded from channels 1 through 32 for the awake (left) and the anesthetized (right) state induced with ketamine-medetomedine. (**C**) We quantified changes in the stability of the models by comparing the distributions of damping time scales (*Reλ*) using a Kolmogorov-Smirnov test. The y-axis corresponds to the KS coefficient of comparing the initial reference distribution (awake, no drugs) versus the distributions of damping time scales obtained at subsequent time stamps.

A total of 16 experiments each consisting of a single pharmacologically-induced reversible loss of consciousness were analyzed in this work. Figure 1A shows the electrode placement and Fig. 1B shows the recordings of channels 1 through 32 (indicated by red dashed lines in Fig. 1A) for a short temporal segment (500 *ms*). As a way to track changes in the dynamical stability (DS) of the fitted VAR(1) models as anesthesia is induced, we compare the initial distribution of damping time scales (*Re*(*λ*)) against subsequent distributions using a Kolmogorv-Smirnov test. Figure 1C shows the KS value of the test along the course of a full experiment in one subject, for each anesthetic. First ($t\, < \,1000\,secs$), the subjects are resting with their eyes open and no drugs are given. At about *t* = 1000 the subjects are blindfolded and this changes the stability of the models. Drugs are given at time $t\,\approx \,2200\,secs$ and responsiveness assessments are performed to determine the state of the subject. The subjects recover from anesthesia (*t* > 5500). Thus, loss of consciousness is associated with significant differences in the distribution of the eigenvalues of the VAR(1) models fit to ECoG signals for both ketamine medetomidine and for propofol.

Here we sought to determine whether the changes in the distribution of the dynamical stability (DS) parameter can be used to predict whether a subject is conscious or not by using a short temporal segment Δ of the ECoG recordings. In order for this prediction to be successful, the differences in the DS distribution in the awake and the anesthetized state must be consistent not just on average but also within short segments of data. In order to test the ability of DS to decode the behavioral state we considered two different validation schemes: within and cross session validation. First, the classifier is trained using the subset of data in a particular session with known behavioral labels (training dataset). The classifier is then used to predict the behavioral state in a validation dataset constructed from the data left out of the training dataset in the same experimental session (within session validation). In the second validation scheme classifier is trained on the data from one experimental session but is validated on the data from a different experimental session for the same drug (cross-session validation). The performance of the classifiers trained on dynamical stability (DS) was compared to those trained on the spectra of the same signals (FFT). Furthermore, we compared the performance of the classifiers trained on stability of real signals to those trained on the stability of phase and stagger surrogates (methods).

**Figure 2:**
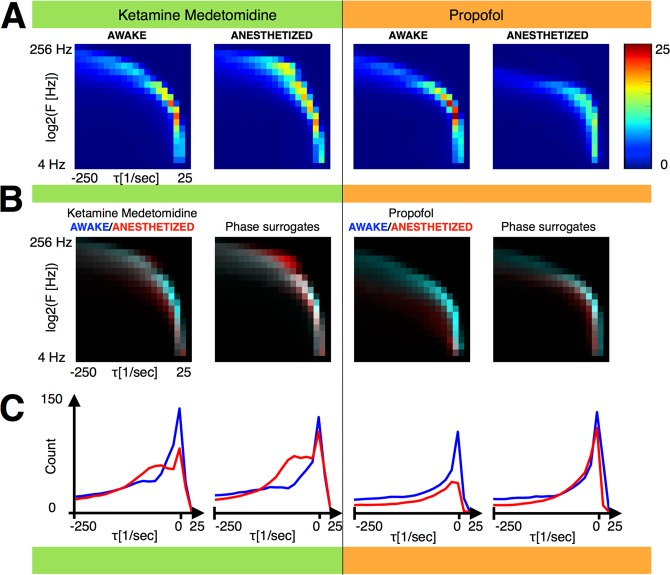
Stabilization of brain dynamics accompanies loss of consciousness. All data in this figure are collected from subject M1 exposed on different occasions to ketamine medetomidine (left two panels highlighted in greeen) or to propofol (right two panels highlighted in brown). **(A)** Distributions of DS parameters of the fitted autoregressive models. These distributions are used to construct vectors (methods) for training and validation of the SVMs. Mean vectors computed over Δ = 2 *s* windows are shown. **(B)** Overlay between awake (cyan) and anesthetized (red) distributions. Histograms were normalized between 0 and 1 and converted to color values such that equal occupancy in the awake and anesthetized states appears in white/cyan, higher prevalence in the awake state appears cyan, higher occupancy in the anesthetized state appears red, and no occupancy in either condition appears black. The lack of appreciable regions of white/cyan color indicate the marked differences in the distributions of the stability parameters between the awake and the anesthetized state. Some differences in the distribution of DS are also evident in models estimated on phase surrogates. **(C)** Distribution of damping timescales *τ* for awake (blue) and anesthetized (red) conditions, for real (left) and surrogate (right) data for each drug combination. To construct the distribution of *τ*, the distributions in **2B** are integrated along the frequency axis for the awake (blue) the anesthetized (red) state. This confirms that loss of consciousness is associated with stabilization of brain dynamics and that this stabilization is much more dramatic for the real rather than surrogate data sets.

Figure 2A shows the distribution of eigenvalues obtained for subject M1 for both drug combinations in the awake and the anesthetized conditions. The differences between distribution of DS in the awake and the anesthetized state for both drug combinations are highlighted in the overlay plots (Fig. 2B). The awake distribution is represented in cyan while the anesthetized distribution is represented in red. The points in which these distributions have similar occupancy are then represented in white/cyan. The paucity of white/cyan in Fig. 2B indicates that the distributions are markedly different in most locations the plane spanned by frequency and damping timescale. Our previous work suggests that loss of consciousness is concomitant with increased stability of the fitted models (more negative damping timescales *τ*). This observation is consistent with the plots shown in Fig. 2C. We performed the same analysis using phase surrogates of the data (see methods). Phase surrogation clearly disrupts the dynamical stability of the fitted models suggesting that the eigenvalues of the fitted autoregressive matrices reflect the correlation structure of the data. Yet, surrogation does not erase the differences in the dynamical stability between the awake and the anesthetized state. This is not entirely surprising as loss of consciousness is associated not just with changes in the correlation structure among different signals but also autocorrelations of each individual signal.

**Figure 3:**
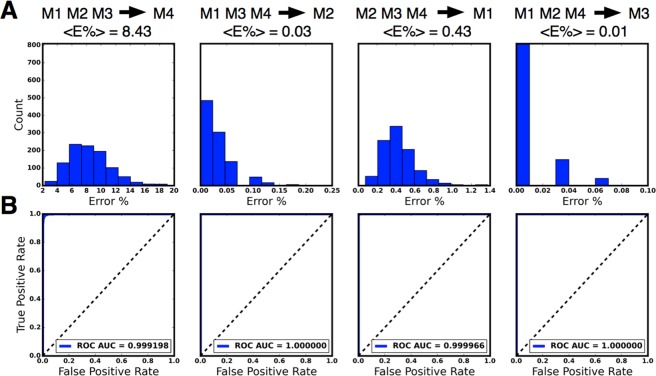
Cross subject validation of the classifier using Ketamine Medetomidine. Each subject was left out of the training dataset in turn. Classifiers were trained on DS vectors averaged over window size Δ = 2000 *ms* from all three remaining subjects. **(A)** Histograms show the error of the classifiers for *N* = 1000 folds replicas of the training and validation data sets. **(B)** Receiver operating characteristic (ROC) curves showing the performance of the classifier as its discrimination threshold is varied. The area under the curve (AUC) is indicated in the figure labels.

Figure 3 shows the results of the cross session cross subject validation where the data from each one of the four subjects was left out of the training dataset. In this case the training (T) set corresponds to stacking all the vectors for the KM condition in three subjects and the validation set (V) is obtained by stacking all the vectors for the KM condition in the left out subject. The vectors were built using Δ = 2 *s*. The figure corresponds to the histograms of scores obtained for *N*_*f*_ = 1000 folds. In order to obtain a measure of the statistical power of the classifier, we computed the ROC curves. This curve computes the tradeoff between false positives and false negatives as the discrimination threshold of the classifier is varied. The area under the curve (AUC) is a measure of the statistical power of the classifier. As *AUC* ≈ 1 across all subjects, given two seconds of recording, the classifier is nearly perfect at distinguishing the awake from the anesthetized state.

**Figure 4:**
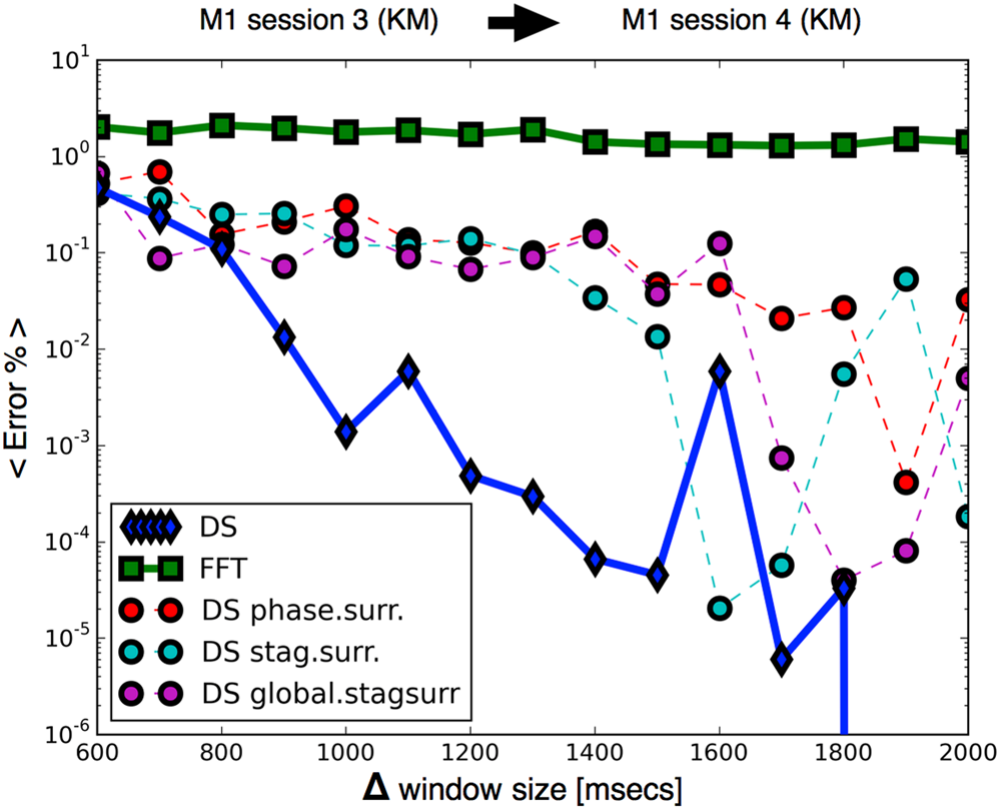
Performance increases as more data is included in the vectors. Improvement in performance for increasing window size Δ across sessions (Ketamine Medetomidine): we test the performance of the protocol for increasing amounts of data that is used to build each vector. We train the classifiers in M1 session 3, and predict the state of the same subject M1 in session 4. The plot compares the performance of the task on vectors built with surrogate data against vectors built with undisrupted data. Dynamical stability (DS) outperforms a local spectral method (FFT) and disruption of global information leads to worse performances. The number of folds at each point is *N*_*f*_ > 10^5^.

**Figure 5:**
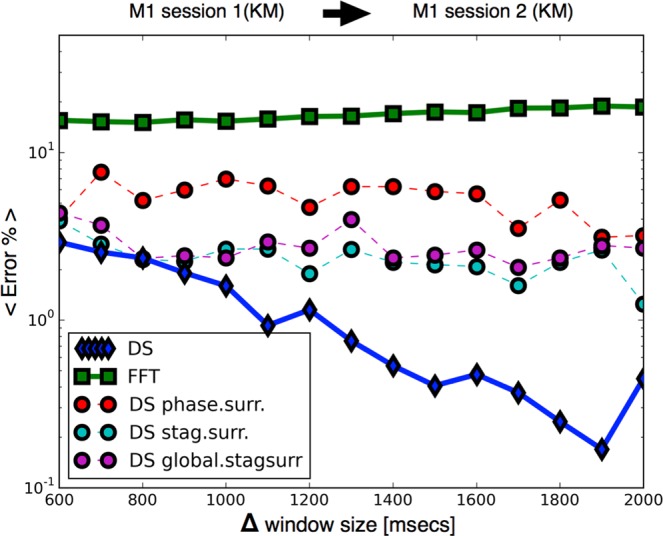
Improvement in performance for increasing window size Δ across sessions (Ketamine Medetomidine): we test the performance of the protocol for increasing amounts of data that is used to build each vector. We train the classifiers in M1 session 1, and predict the state of the same subject M1 in session 2. The number of folds at each point is *N*_*f*_ > 10^4^.

Figure 4 shows the performance of the classifier as more data is utilized to build the Ketamine Medetomidine vectors. We performed this test by training the classifiers with vectors from one session recorded in subject M1 (T), and then tested our predictions on a validation dataset (V) from M1 receiving ketamine medetomidine anesthesia on a different day. As Δ grows the performance of the classifier based on dynamical stability (DS) grows until finite data size effects become significant and result in fluctuations in performance. In contrast, the performance of the classifier built using the spectral characteristics of the same data (FFT) or in three types of data surrogates is not significantly improved by increasing Δ. Overall, given one second of data DS-based classifier outperforms the next based classifier by 2 orders of magnitude and outperforms spectral-based classifier by 3 orders of magnitude. The benefit of DS-based classifier is more modest when different sessions were used as training and validation datasets (Fig. 5). Nevertheless DS-based classifier outperforms both the classifier based on spectral estimates and on data surrogates.

**Figure 6:**
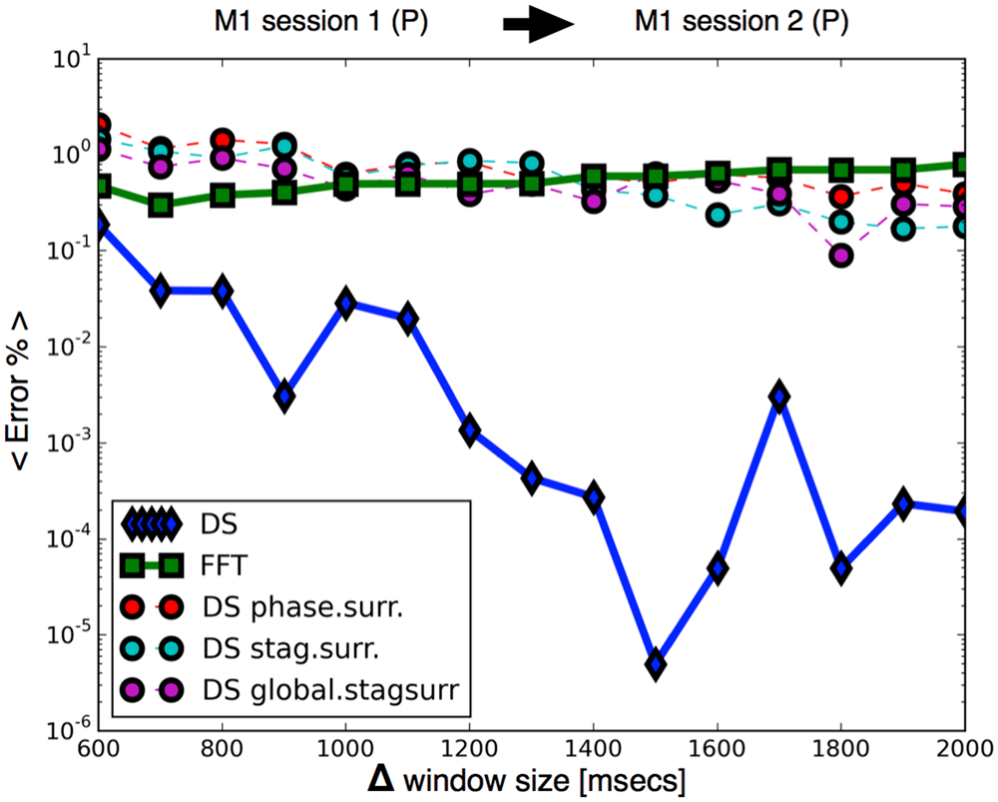
Improvement in performance for increasing window size Δ across sessions (Propofol). Dynamical stability (DS) outperforms a local spectral method (FFT) and disruption of global information leads to worse performances which are similar to (FFT). The number of folds at each point is *N*_*f*_ > 10^4^.

In Fig. 6 we show the same analysis as in Figs 4 and 5 for inductions with Propofol across sessions for subject *M*1. In this case, the performance of the classifiers on the (DS) vectors is about 3 to 4 orders of magnitude better than on the (FFT) vectors. Interestingly, in this case the (FFT) vectors perform similarly to the (DS) vectors built with surrogate data. This is also consistent with Fig. 1 row C in which we show that while in the Ketamine Medetomidine case, bulk differences across states persist after surrogation, for the case of Propofol differences across states depend more strongly on global correlations which are disrupted by surrogation. Performance across subjects for propofol is not presented in this work since our analysis suggests that M4 does not become fully anesthetized under the observed experimental conditions. This in turn could be due to the fact that M4 belongs to a different species than the other subjects^24^.

We found that the performance of the classifier trained on real data is significantly better than the classifiers trained on surrogate data or on spectra of signals for both ketamine medetomidine and for propofol. This suggests the exciting possibility that the classifier is picking up on global dynamical features which are disrupted by surrogation. Our results suggest that a simple linear approach is able to capture global features that are associated to loss of consciousness. Our numerical estimates provide a measure of the utility of such global information. Consistent with the fact that the stability of the fitted models is to a certain degree independent of global correlations, the performance of our procedure is only moderately worsened by disruption of such global features. This suggests that high performances might be achieved by these means even in the more common situation in which a smaller portion of the cortex is being monitored

## Conclusions

Unveiling the mechanisms by which consciousness emerges is among the ultimate goals of systems neuroscience. Within this broad scope, a more immediate goal is understanding the quantitative imprint of consciousness on electrophysiological activity. Our efforts herein are aimed at establishing a relationship between brain activity under different anesthetic regimes and dynamical stability of cortical activity. To establish this relationship we performed linear stability analysis of electrophysiological recordings acquired using ECoG in non human primates during reversible suppression of consciousness induced with mechanistically distinct anesthetics. Our previous studies have shown that when consciousness is extinguished by anesthetics, dynamical stability (DS) of the ECoG signals is increased. Thus, the brain becomes less responsive. This stabilization is reversed upon re-emergence of consciousness^24,25^. Here we took advantage of this stabilization to demonstrate how DS can be used to reliably distinguish between activity in the awake and the anesthetized brain.

Our previous results^24^ suggest that the distribution of DS is remarkably consistent across time within the same subject during the awake state. This suggests, that deviation from this time-independent distribution could be used as a measure of deviation from the awake state. Here, we tested this possibility by training SVMs to distinguish between the awake and the anesthetized state. We confirmed that, given a short (≤1 *s*) temporal window, distribution of DS reliably distinguishes between the awake and the anesthetized state. We also extend our previous findings by demonstrating that the distribution of DS is not only consistent in time within the same individual but is also consistent between individuals. This consistency is what enables the near perfect cross subject classification in Fig. 3.

Many other measures of neuronal activity are known to change when consciousness is lost^1,2,4–6,10,13,14,32^ and others. While exhaustive comparison to all previously used measures of neuronal activity is not practical, here we explicitly compared performance of the classifier trained on DS to that trained of spectral features of the same signals. Changes in neuronal oscillations captured by the spectral analysis are amongst the oldest and the most commonly used measures of brain activity used to distinguish between states of consciousness. This comparison revealed that classification based on DS is several orders of magnitude superior to that based on spectral features. This suggests that DS is more consistently altered during loss of consciousness than the spectral characteristics of the signals. Furthermore, while the classification based on DS improved with increasing the temporal window over which measurements were averaged, there was no marked increase in classification accuracy based upon the spectral features or on the staggered surrogates of the data. This suggests that linear stability analysis of the ECoG signals reflects not just the properties of individual ECoG recordings but collective behavior that arises out of the interactions among different cortical regions that give rise to ECoG signals. It is possible that anesthetic-induced changes in functional connectivity^6,10,33–35^ and response complexity^11,13,14^ may be closely related to the loss of dynamical criticality suggested by the linear stability modeling.

The assumptions behind our procedure are minimal, namely, that the dynamics is approximately linear in short temporal intervals. This assumption enables efficient estimation procedures and we kept the simplest approach within this premise. This simplicity allowed us to estimate DS in short time windows and still attain high classification accuracy. The ability to classify short segments has significant advantages for inferring the state of consciousness on the basis of brain activity. For instance, a key distinction between minimally conscious and vegetative state is that the former is characterized by sporadic and sometimes brief episodes of awareness. This complicates clinical diagnosis of minimally conscious state^36^. It has been known since the 1970s that some patients become inadvertently briefly aware under anesthesia^37–40^. Furthermore these episodes of awareness are not reliably picked up by the current clinically used depth of anesthesia monitors^38–40^ even when awareness is associated with recall^37^. The performance of the classifiers trained on DS is several orders of magnitude better than clinically used monitors designed to detect awareness under anesthesia. For instance Zand *et al*.^41^, studied the specificity and sensitivity of the bispectral index–the most widely used depth of anesthesia monitor–for detecting episodes of awareness under anesthesia for Cesarean sections. They found that the sensitivity and specificity of the bispectral index was 68.8% and 57.3% respectively. Furthermore, Schneider *et al*.^42^ were not able to find statistically significant differences between bispectral index values in patients who showed behavioral evidence of awareness under anesthesia and those that did not^43–61^.

While the dramatic improvement in the classification ability of the DS-based detection of consciousness relative to the existing clinically-used methods is encouraging, clearly the approach in the present manuscript cannot be directly applied to patients because our classifier was trained on ECoG rather than EEG recordings. These invasive recordings are not routinely available in patients undergoing surgery or those suffering from disorders of consciousness. It is very likely that our ability to estimate DS from non-invasive EEG recordings will be decreased relative to the ECoG and the classification ability will degrade somewhat. It is also likely that additional processing such as artifact rejection will be required to adapt our methodology to clinically available EEG recordings acquired in an electrically noisy operating room. That being said, given the same quality of recordings, spectrum-based and surrogate-based classifiers were significantly worse than those trained on DS. This suggests that our superior ability to decode whether the subject was conscious or not is not solely due to the high quality of the recordings. Further work will also be needed to determine whether DS-based classification works reliably for other kinds of anesthetics not used in this study as well as behavior of the stability analysis in sub-anesthetic drug concentrations.

## Data Availability

The datasets generated during and/or analysed during the current study are available from YT on reasonable request. See http://neurotycho.org/ for partial access to this dataset.
