# Author Correction: Single-trial classification of awareness state during anesthesia by measuring critical dynamics of global brain activity

**DOI:** 10.1038/s41598-019-55180-0

**Published:** 2019-12-04

**Authors:** Leandro M. Alonso, Guillermo Solovey, Toru Yanagawa, Alex Proekt, Guillermo A. Cecchi, Marcelo O. Magnasco

**Affiliations:** 1 Laboratory of integrative neuroscience, The Rockefeller University, New York, NY 10065 USA; 2 Present Address: Volen Center for Complex Systems, Department of` Biology, Brandeis University, Waltham, MA 02454 USA; 3 Instituto del Cálculo, FCEyN, Universidad de Buenos Aires, (C1428EGA), Buenos Aires, Argentina; 4 Laboratory for Adaptive Intelligence, Brain Science Institute, RIKEN, Saitama, 351-0198 Japan; 5 Anesthesiology and Critical Care, University of Pennsylvania, Philadelphia, PA 19104 USA; 6 IBM, Thomas J. Watson Research Center, Yorktown Heights, NY USA

Correction to: *Scientific Reports* 10.1038/s41598-019-41345-4, published online 20 March 2019

In the original version of this Article, Leandro M. Alonso and Guillermo Solovey were omitted as equally contributing authors. This has now been corrected in the PDF and HTML versions of the paper.

